# A PRISMA-based systematic review on advances in identity recognition and authentication using human biometric signals (2018–2023)

**DOI:** 10.1186/s12938-025-01508-z

**Published:** 2026-01-02

**Authors:** Bahadır Çokçetin, Muhammed Kürşad Uçar

**Affiliations:** 1 Faculty of Engineering, Electrical-Electronics Engineering, Sakarya University, Serdivan, 54187 Sakarya Turkey; 2 MKU Technology, Serdivan, Sakarya Turkey

**Keywords:** PRISMA, Systematic review, Biometric signals, Authentication, Recognition, Identification

## Abstract

This systematic review examines the effectiveness of physiological biometric signals in authentication and recognition systems by analyzing studies published between 2018 and 2023. Specifically, different biometric modalities (e.g., ECG, EEG, and PPG), commonly used datasets, signal processing techniques, and classification approaches are evaluated to assess their reported reliability and performance. In addition, the performance of multimodal biometric systems is compared with that of unimodal approaches. The review was conducted in accordance with the PRISMA 2020 guidelines. Relevant studies published between 2018 and 2023 were systematically retrieved from major databases, including EBSCO, PubMed, IEEE Xplore, Scopus, and Web of Science. A total of 2,064 records were initially identified, and after duplicate removal and eligibility screening, 80 articles were included in the final review. The study selection process is summarized using a PRISMA flow diagram. The reviewed studies indicate that ECG-based authentication systems report high average accuracy (98.6%), while multimodal biometric systems generally achieve accuracy levels exceeding 99%. Across modalities, deep learning–based approaches tend to outperform traditional machine learning methods. Dataset size and the choice of signal processing techniques were also found to influence reported performance outcomes. Overall, the findings suggest that biometric signal–based authentication systems demonstrate strong performance under the evaluation conditions reported in the literature. Multimodal fusion and deep learning approaches appear particularly promising, although reported results vary across datasets and protocols. Future research should prioritize larger and more diverse datasets, standardized evaluation benchmarks, and optimized signal processing pipelines to improve comparability and real-world applicability. Further studies on the integration of complementary biometric signals are also warranted.

## Highlights

1. The performance of biometric signal-based authentication systems was analyzed. The study examined how biometric signals such as ECG, EEG, and PPG are used in authentication systems.
2. The highest accuracy rate was achieved with ECG-based systems. ECG-based systems were found to be the most reliable biometric method, with an average accuracy rate of 98.6%.
3. Deep learning models achieved accuracy rates up to 99.3% (EEGMMIDB dataset) and 99.8% (ECG PTB-XL dataset), consistently outperforming traditional machine learning methods (typically 95–97%).
4. Multimodal biometric systems were found to be more reliable than unimodal systems. Mul timodal biometric systems were considered more robust and resistant to attacks than single signal-based methods.
5. Larger datasets and advanced signal processing techniques were associated with improved accuracy. The size of the dataset, signal processing methods, and classification algorithms directly impacted the performance of biometric authentication

## Introduction

Conventional authentication mechanisms such as passwords, PIN codes, and smart cards suffer from well-known shortcomings: they can be forgotten, guessed, disclosed, or intercepted by adversaries [1]. In contrast, biometric authentication relies on unique physiological and behavioral characteristics that are inherently bound to the individual, thereby reducing the attack surface and improving usability [2, 3]. Within this landscape, physiological signals have attracted growing interest because they inherently contain liveness cues and are more resistant to large-scale spoofing. Biometric authentication has thus shifted from niche applications to broader deployment across consumer, clinical, and industrial settings [4, 5].

Among physiological traits, electrocardiogram (ECG), electroencephalogram (EEG), and photoplethysmogram (PPG)—including its contactless variant rPPG—have emerged as key modalities for personal recognition and identity verification. Their appeal lies in their distinctiveness, temporal stability, and increasing availability through wearable and imaging sensors. [6, 7]. Biosignal authentication is now applied across multiple domains—healthcare (continuous patient monitoring, access control), mobile and IoT devices (smartwatch- or camera-based authentication), and automotive systems (driver identification)—demonstrating their practicality beyond controlled laboratory environments [8–10]. Despite this progress, significant challenges remain in achieving robustness, scalability, and cross-modality standardization.

Prior reviews have typically focused on single modalities, leaving cross-modality comparisons and unified evaluation frameworks largely underexplored [5, 11]. Methodological heterogeneity, lack of standardized metrics (AUC, EER), and small-sample evaluations limit direct comparability and reduce generalizability [7, 12]. Technical challenges—such as noise sensitivity in EEG, motion artifacts in PPG/rPPG, and inter-session variability in ECG—remain key barriers to deployment [13, 14]. Deep learning models (CNN, RNN, Transformer) outperform handcrafted approaches but require large, diverse datasets and robust domain adaptation strategies to maintain long-term stability [15, 16].

An emerging trend is multimodal fusion, in which complementary signals (e.g., ECG with PPG or EEG with face) achieve superior accuracy (99.3–99.8%) [15]. Parallel advances in federated and privacy-preserving learning enable decentralized model training without requiring raw data sharing, aligning biometric research with regulations such as GDPR [17–19]. Beyond technical aspects, user acceptance, ethical considerations, and transparent dataset governance are essential for ensuring trust and broad adoption [20, 21].

*Contributions:* This study presents a PRISMA-guided systematic review of biometric authentication based on physiological signals (2018–2023). We (i) synthesize evidence across ECG, EEG, PPG/rPPG, EMG/sEMG, face, and fingerprint to enable cross-modality comparison; (ii) quantify performance through subgroup analyses by dataset size and methodological approach; (iii) distinguish verification (1:1) and identification (1:N) tasks and emphasize the need for standardized reporting (e.g., AUC, EER); and (iv) examine security-oriented designs including encryption and privacy-preserving strategies.

*Research questions:*

1. Which physiological signals (e.g., ECG, EEG, PPG/rPPG) achieve the most reliable performance in biometric *verification* and *identification*, and under what conditions?
2. How do preprocessing pipelines and classification paradigms (traditional ML vs. deep learning) influence accuracy and robustness?
3. What benefits do multimodal fusion and privacy-preserving solutions provide relative to unimodal baselines?
4. What methodological gaps (e.g., cross-session variability, dataset scale, or metric standardization) remain, and how can future studies effectively address them?

*Article organization:* Section "Materials and Methods" details the search strategy and quality assessment. Section "Results" reports modality-wise findings; Section "Discussion" interprets the findings in the context of existing literature, and Section "Conclusion and Recommendations" concludes with recommendations for future work.

## Materials and methods

### Literature search strategy

This systematic review conducted a comprehensive literature search on biometric-based identity recognition and authentication. The search, completed in December 2023, covered studies published between 2018 and 2023 in major databases such as EBSCO, PubMed, IEEE Xplore, Scopus, and Web of Science.

The keywords ‘biometric authentication’, ‘biometric recognition’, and ‘biometric identification’ were combined using Boolean operators (AND, OR) to maximize retrieval sensitivity. Additionally, reference lists of relevant studies were screened to identify further eligible works.

A total of 2,064 records were identified across the five databases and additional sources. After removing 13 duplicates, 2,051 records remained for screening. Of these, 919 were excluded as unrelated to human biometric data, and 1,132 proceeded to full-text review. Fifty-five review papers and four studies lacking accuracy metrics were subsequently excluded, leaving 80 eligible articles focused on biometric signal processing. Ultimately, 80 articles met the inclusion criteria, involving biometric signal processing techniques applied to human biometric data. These articles constituted the final dataset for systematic evaluation.

The complete search strategies for each database are provided in the Supplementary Materials (Table 14). For example, in PubMed, the following search string was used: (‘biometric authentication’ OR ‘biometric recognition’ OR ‘biometric identification’) AND (‘ECG’ OR ‘EEG’ OR ‘PPG’), limited to English-language publications from 2018 to 2023.

### Inclusion and exclusion criteria

**Fig. 1:**
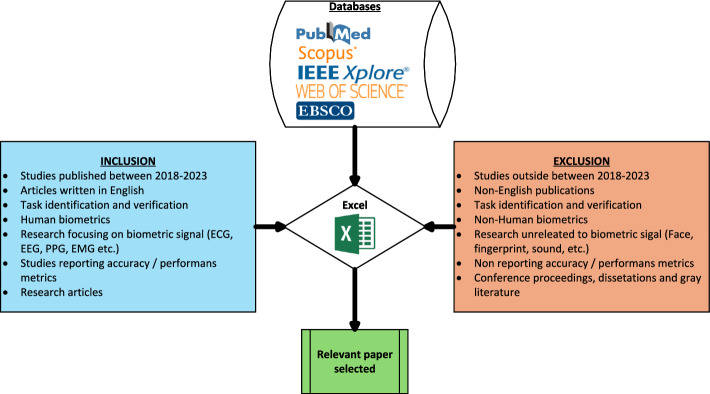
Study selection process according to the PRISMA 2020 guidelines

To ensure methodological consistency and reliability, only peer-reviewed journal articles were included, whereas conference proceedings, dissertations, and gray literature were excluded. Studies were included if they were published between 2018 and 2023, written in English, and focused on biometric signals (e.g., ECG, EEG, or PPG) used for authentication or recognition. Additionally, included studies were required to report accuracy or other relevant performance metrics. Conversely, studies were excluded if they were published outside the 2018-2023 timeframe, written in languages other than English, or unrelated to biometric signal authentication. In addition, conference papers, dissertations, and other forms of gray literature were systematically excluded. Figure 1 provides a concise summary of the inclusion and exclusion criteria.

### Data collection process

After completing the literature search, all articles meeting the inclusion criteria were reviewed in full. In the first phase, titles and abstracts were screened, and eligible studies were selected for full-text review.

In the second phase, full-text screening identified studies on identity recognition and authentication using human biometric signals that met the criteria. Data extracted included publication year, biometric signal type, classification methods, dataset size, and reported accuracy rates. All extracted data were tabulated in Microsoft Excel.

Datasets used in the reviewed studies were tabulated, showing the dataset type and corresponding article title (Table 4). Additionally, Table 3 presents the signal types, datasets used, and corresponding article titles.

### Data items and categories

The collected data were categorized into groups according to biometric signal type, classification method, datasets, and performance metrics. Specifically, the categories included biometric signal (ECG, PPG, EMG), classification methods (SVM, LDA, KNN), datasets employed, and reported accuracy rates. Additional data items included the journal of publication and the number of participants in each dataset. These categories provided the basis for subsequent subgroup analyses.

### Study selection using process PRISMA flow diagram

**Fig. 2:**
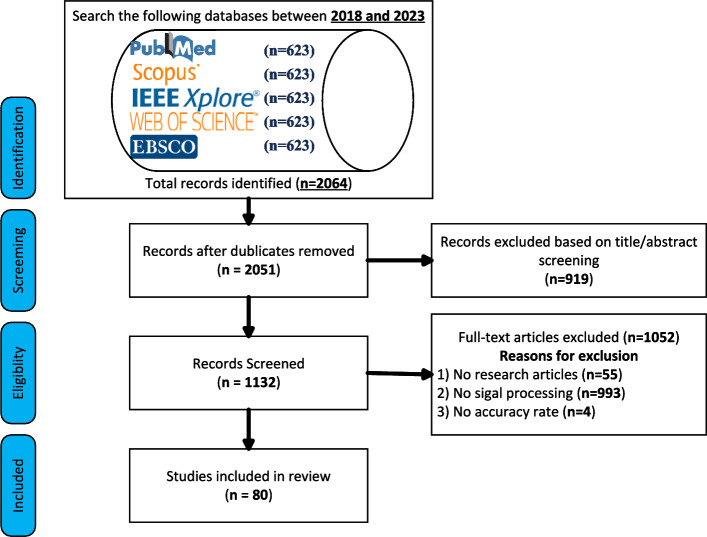
PRISMA flow diagram

The PRISMA flow diagram illustrates the number of studies initially identified, those excluded after title and abstract screening, and those included for full-text review. The diagram also indicates the final number of included studies and the reasons for exclusion (Figure 2).

### Meta-analysis

A formal meta-analysis was not conducted in this review due to substantial heterogeneity among the included studies. Variations in dataset characteristics, preprocessing, feature extraction, and classification pipelines prevented direct statistical pooling. In addition, the lack of standardized accuracy metrics across studies further limits the feasibility of quantitative aggregation.

To provide a valid quantitative overview without introducing bias, we instead conducted subgroup analyses (e.g., by dataset size and methodological approach). These analyses highlight consistent performance trends and serve as a rigorous alternative to a meta-analysis under heterogeneous conditions.

Future research could may enable valid meta-analyses, particularly for ECG and EEG, if standardized benchmark datasets, evaluation metrics such as AUC and EER, and harmonized study designs are adopted. Establishing common performance indicators would make comprehensive meta-analyses feasible in subsequent systematic reviews.

Nevertheless, to provide an indicative quantitative perspective, a summary of reported accuracies across the included studies was calculated. Across 80 studies, the mean reported accuracy was 97.9% (SD = 2.6%), with a median of 99.0% and an interquartile range of 96.3–99.8%. When grouped by modality, ECG-based systems achieved a pooled mean of 98.6%, EEG-based systems 96.8%, and PPG-based systems 98.4%. Although formal confidence intervals were not computed owing to heterogeneous metrics, these descriptive aggregates provide a concise meta-analytic overview of central performance trends (Table 1).

**Table 1:** Descriptive summary of reported accuracy rates across modalities (2018–2023)

Modality	Mean accuracy (%)	Median (%)	Range (%)
ECG	98.6	99.2	91.3–100.0
EEG	96.8	98.5	83.2–100.0
PPG/rPPG	98.4	99.5	95.6–99.9
EMG/sEMG	94.6	96.0	93.1–96.0
Multimodal	99.5	99.7	98.0–100.0
**Overall (all studies)**	**97.9**	**99.0**	**83.1–100.0**

Values represent descriptive averages across 80 included studies. Formal meta-analysis was not conducted due to methodological heterogeneity

### Quality assessment

The methodological quality of the included studies was assessed using the Joanna Briggs Institute (JBI) Critical Appraisal Checklist for Analytical Cross-Sectional Studies. Eight items were evaluated (e.g., clarity of inclusion criteria, validity of measurement methods, completeness of outcome reporting), each scored 0 or 1. Total scores ranged from 0–8, with ≥6 indicating high quality and <6 lower quality. Two reviewers independently performed the assessment, and discrepancies were resolved through discussion. A summary of representative high-performing studies is provided for illustration, while the complete quality appraisal results of all 80 studies are reported in the two-part JBI Quality Assessment tables (Tables 15 and 16).

## Results

### Characteristics of included studies

#### Yearly distribution of publications and source journals

A total of 80 articles published between 2018 and 2023 were included, representing a broad range of journals and publishers. Major publishers included MDPI, Springer, Hindawi, IEEE, and Wiley (Table 2).

MDPI contributed the largest number of studies, with 37 articles from the journal Sensors alone, underscoring MDPI’s strong contribution to biometric-signal research. Springer also holds a prominent position, publishing key studies in Neural Computing and Applications, Circuits, Systems and Signal Processing, and Soft Computing.

Publication output peaked in 2022 (20 articles), reflecting the rapid growth of research interest in biometric signals. The following year, 2021, also showed strong productivity (17 articles).

In addition to MDPI and Springer, IEEE journals contributed significantly, particularly in technology-focused applications of biometric signals. Publishers such as Wiley and Taylor & Francis have also published high-quality research on biometric signals for authentication and recognition. A single article published in TÜBİTAK Academic Journals represents a regional academic contribution.

Overall, these findings highlight the strong international interest in biometric signal research and the broad support provided by publishers.

**Table 2:** Publishing houses, journals, and years with references

Year	Publisher	Journal	Number of articles	References
2018	Springer	Circuits, Systems, and Signal Processing	1	[22]
2018	Hindawi	Computational Intelligence and Neuroscience	1	[23]
2018	MDPI	Sensors	6	[24–29]
2018	PLOS	PLOS ONE	1	[30]
2018	IEEE	IEEE Transactions on Pattern Analysis and Machine Intelligence (TPAMI)	1	[32]
2019	Hindawi	Computational Intelligence and Neuroscience	1	[33]
2019	MDPI	Sensors	4	[34–37]
2019	Springer	Journal of Medical Systems	1	[38]
2019	TÜBİTAK	Turkish Journal of Electrical Engineering & Computer Sciences	1	[39]
2020	Hindawi	Journal of Electrical and Computer Engineering (JECE)	1	[40]
2020	IEEE	IEEE Transactions on Computer-Aided Design of Integrated Circuits and Systems (TCAD)	1	[41]
2020	MDPI	Sensors	5	[42–46]
2020	Nature	Scientific Reports	1	[47]
2020	Springer	The Journal of Supercomputing	1	[48]
2020	Springer	Neural Computing and Applications	1	[49]
2020	World Scientific	Journal of Mechanics in Medicine and Biology	1	[50]
2020	Springer	Multimedia Systems	1	[51]
2021	Hindawi	Computational and Mathematical Methods in Medicine	1	[52]
2021	Hindawi	Scientific Programming	1	[53]
2021	IEEE	IEEE Transactions on Consumer Electronics	1	[54]
2021	IEEE	IEEE Transactions on Instrumentation and Measurement	2	[55, 56]
2021	MDPI	Entropy	1	[57]
2021	MDPI	Sensors	6	[58–64]
2021	Nature	Scientific Reports	1	[65]
2021	Taylor & Francis	Applied Artificial Intelligence	1	[66]
2021	Wiley	Security and Privacy	1	[67]
2021	Wiley	International Journal of Communication Systems	1	[68]
2022	Hindawi	Computational Intelligence and Neuroscience	1	[69]
2022	Hindawi	Scientific Programming	1	[70]
2022	IAENG	Engineering Letters	1	[71]
2022	IOS Press	Journal of Intelligent & Fuzzy Systems	1	[72]
2022	MDPI	Mathematics	1	[73]
2022	MDPI	Sensors	7	[13, 31, 74–79]
2022	MDPI	Diagnostics	1	[80]
2022	Springer	Cybernetics and Systems Analysis	1	[81]
2022	Springer	Multimedia Systems	1	[51]
2022	Taylor & Francis	IETE Journal of Research	1	[82]
2022	Taylor & Francis	Journal of Discrete Mathematical Sciences and Cryptography	1	[83]
2022	Wiley	Security and Privacy	1	[84]
2022	PubMed (index)	Sensors (Basel)	1	[85]
2023	MDPI	Behavioral Sciences	1	[86]
2023	MDPI	Diagnostics	1	[80]
2023	MDPI	Information	1	[87]
2023	MDPI	Sci	1	[15]
2023	MDPI	Sensors	7	[88–94]
2023	Springer	Neural Computing and Applications	1	[95]
2023	Springer	Soft Computing	1	[10]
2023	Taylor & Francis	Computer Methods in Biomechanics and Biomedical Engineering	1	[96]
2023	Taylor & Francis	IETE Journal of Research	1	[97]

Publishers and journal titles are presented as reported in the included studies; minor style harmonizations applied for consistency (e.g., capitalization, diacritics)

#### Biometric datasets and sample sizes

This section examines studies using different biometric signal datasets. Table 4 summarizes the signal types, datasets, participant numbers, and reported accuracy rates. This analysis explores how dataset characteristics influence the accuracy of different biometric signals (Table 4).

ECG was the most frequently used signal type among the reviewed studies. Large datasets such as PTB-XL and PhysioNet CinC 2017 consistently yielded accuracy rates above 98%, confirming the robustness of ECG-based authentication. EEG-based studies exhibited variable accuracy across datasets (86–97.8%; e.g., EEGMMIDB). The inherent complexity of EEG signals likely contributes to this variation.

PPG datasets such as Real-World PPG and MIMIC-II achieved accuracy rates above 98 %, supporting their suitability for heart rate- and blood flow-based biometric systems. EMG-based systems, which rely on muscle-activity patterns, generally reported accuracy around 96%.

Face- and fingerprint-based studies reported accuracy rates of 94–98%, with FERET and VeinPolyU datasets achieving the highest performance. The table highlights which signal types and datasets provide better performance based on accuracy comparisons.

**Table 3:** Articles and datasets used across modalities

Signals	Datasets	Subjects	Accuracy (%)	References	Signals	Datasets	Subjects	Accuracy (%)	References
ECG	ECG-ID	90	99.85	[87]	ECG	MIT-BIH Multi-parameter	18	100.00	[10]
ECG	MWM-HIT, ECG-ID	90	99.89	[15]	ECG	Personal Measurement	150	99.80	[24]
ECG	PTBDB	290	95.30	[82]	ECG	MIT-BIH Arrhythmia	47	95.46	[39]
ECG	Personal Measurement	18	99.87	[84]	ECG	MLII, UCI Arrhythmia, PTBDB	290	100.00	[65]
ECG	DREAMER	23	91.30	[55]	ECG	PhysioNet QT	22	94.16	[42]
ECG	PTB Diagnostic	115	99.00	[88]	ECG	PhysioNet Computing in Cardiology 2018	1985	92.00	[43]
ECG	Personal Measurement	100	95.40	[58]	ECG	FANTASIA, MIT-BIH, CYBHi	200	100.00	[44]
ECG	ECG-ID	90	99.05	[59]	ECG	MIT-BIH Arrhythmia	47	99.00	[96]
ECG	PhysioNet ECG-ID	20	99.13	[48]	ECG	MIT-BIH Normal Sinus Rhythm, MIT-BIH Arrhythmia	50	99.80	[45]
ECG	PTBDB	290	99.69	[80]	ECG	Personal Measurement	11	87.61	[60]
ECG	APNEA-ECG, LTAFDB, MITDB, LTDB, VFDB, SLPDB, SVDB, INCARTDB, FANTASIA, PTB-XL	500	98.00	[74]	ECG	European ST-T	79	99.14	[90]
ECG	Personal Measurement	18	93.14	[27]	ECG	ECG-ID	90	94.00	[71]
ECG	Schiller ECG Database	460	98.40	[28]	ECG	ECG-ID, MIT-BIH, NSR-DB	156	99.62	[91]
ECG	Personal Measurement	17	100.00	[76]	ECG	Personal Measurement	55	99.30	[29]
ECG	CYBHi	63	98.42	[77]	ECG	PTBDB, CUECG	390	98.10	[36]
ECG	TW, VeinPolyU; MWM-HIT, ECG-ID	90	98.60	[51]	ECG	Personal Measurement	20	99.00	[62]
ECG	Schiller ECG Database	460	97.50	[30]	ECG	Personal Measurement	3133	99.60	[81]
ECG	Personal Measurement	100	98.00	[46]	ECG	PTBDB, CYBHi	290	99.27	[67]
ECG	FANTASIA, MIT-BIH Arrhythmia, ECG-ID, European ST-T, Aveiro ECG	200	98.00	[56]	ECG	PhysioNet Computing in Cardiology Challenge 2017	50	99.30	[78]
ECG	MIT-BIH Arrhythmia	47	95.17	[79]	ECG	PTBDB and ECG-ID	290	99.90	[57]
ECG	PhysioNet ECG-ID, PhysioNet QT, PhysioNet NSRDB	183	98.31	[37]	ECG	ECG-ID, PTBDB, CEBSDB	400	100.00	[41]
EEG	Personal Measurement	20	98.50	[54]	EEG	EEGMMIDB	109	83.21	[33]
EEG	Personal Measurement	16	97.17	[66]	EEG	EEGMMIDB	109	100.00	[95]
EEG	WAY_EEG_GAL	12	83.15	[13]	EEG	Personal Measurement	42	97.60	[23]
EEG	EEGMMIDB	109	98.54	[73]	EEG	EEGMMIDB	109	93.86	[69]
EEG	EEGMMIDB	109	98.80	[38]	EEG	Personal Measurement	39	91.10	[89]
EEG	BCI Competition 2008—Graz A	9	96.00	[83]	EEG	EEGMMIDB	109	99.98	[22]
EEG	Personal Measurement	45	94.27	[26]	EEG	EEGMMIDB	109	99.00	[98]
EEG	RSVP, Sternberg Task, BCI2000	139	99.00	[86]	EEG	EEGMMIDB	109	99.00	[50]
EEG	MAHNOB-HCI	35	99.10	[52]	EEG	Personal Measurement	58	98.78	[35]
EEG	BED	21	86.74	[85]	EEG	Personal Measurement	29	96.70	[92]
EEG	Personal Measurement	20	88.00	[49]	EEG	EEGMMIDB	109	100.00	[94]
EEG	EEGMMIDB	109	97.00	[63]	EEG	EEGMMIDB	109	99.00	[99]
EEG	EEGMMIDB	109	99.30	[47]	EEG	Personal Measurement	21	99.00	[31]
EMG	Personal Measurement	8	96.00	[97]	PPG	Personal Measurement	23	95.65	[34]
PPG	PRRB, MIMIC-II, Berry–Nonin	200	99.00	[25]	PPG	Real-World PPG	35	99.50	[68]
PPG	BIDMC, MIMIC, CapnoBase	127	99.75	[70]	PPG	Real-World PPG	35	99.00	[93]
PPG	BIDMC, MIMIC, CapnoBase	127	99.70	[40]	PPG	BIDMC, MIMIC, CapnoBase	127	99.88	[72]
PPG	BIDMC, MIMIC, CapnoBase	127	99.69	[53]	PPG	Real-World PPG	35	97.00	[64]
rPPG	Personal Measurement	17466	99.74	[75]	sEMG	Personal Measurement	4	93.10	[61]

datasets and accuracies are reported as stated by the original authors (best result per article when multiple results exist). “Personal Measurement” denotes subject-specific datasets collected by the study authors; subject counts refer to the corresponding article and may differ from full dataset sizes

#### Datasets and articles used

Table 4 summarizes the datasets employed in the reviewed studies, indicating the corresponding articles and citations.

A summary of the biometric datasets used in the reviewed studies, including the number of participants and reported accuracy rates, is provided in Table 3. The most frequently used datasets included EEGMMIDB (for EEG), PTB-XL and PhysioNet ECG-ID (for ECG), and BIDMC (for PPG). Dataset sizes varied substantially, from small-scale studies (e.g., 7 participants in RSVP) to large-scale studies (e.g., 17,466 participants in rPPG-based research).

Reported accuracy varied notably depending on dataset characteristics and preprocessing techniques. For instance, studies using ECG-ID report accuracy rates of 99.2–99.5%, while EEG-based studies exhibit a broader range (83.15%–100%). These findings highlight the impact of dataset size and preprocessing methods on biometric authentication performance. Further methodological details, including preprocessing and feature-extraction techniques, are summarized in Tables 5 and 6.

**Table 4:** Datasets and articles used

Signals	Dataset name	Dataset short name	Dataset citation	Subjects	Article used
ECG	APNEA-ECG	APNEA-ECG	[100]	35	[74]
ECG	Aveiro ECG	Aveiro	N/A	10	[56]
ECG	Cardiology Challenge 2017	Cardiology 2017	[100]	50	[78]
ECG	Cardiology Challenge 2018	Cardiology 2018	[100]	1985	[43]
ECG	CUECG	CUECG	[36]	100	[36]
ECG	Check Your Biosignals Here	CYBHi	[101]	63	[44, 67, 77]
ECG	DREAMER	DREAMER	[102]	23	[55]
ECG	ECG-ID	ECG-ID	[100]	90	[15, 37, 41, 48, 51, 56, 57, 59, 71, 87, 91]
ECG	European ST-T	European	[100]	79	[56, 90]
ECG	FANTASIA	FANTASIA	[100]	40	[44, 56]
ECG	St Petersburg Arrhythmia	INCARTDB	[100]	75	[74]
ECG	Long Term AF	LTAFDB	[100]	84	[74]
ECG	MIT-BIH Long-Term ECG	LTDB MIT-BIH	[100]	7	[74]
ECG	MIT-BIH	MIT-BIH	[100]	36	[44, 91]
ECG	MIT-BIH Multi-parameter	MIT-BIH-MP	[100]	18	[10]
ECG	MIT-BIH Arrhythmia	MITDB	[100]	47	[39, 45, 56, 79, 96]
ECG	MLII	MLII	[100]	47	[65]
ECG	MWM-HIT ECG	MWM-HIT	[103]	70	[15, 51]
ECG	MIT-BIH Normal Sinus Rhythm	MIT-BIH NSR	[100]	18	[37, 45]
ECG	NSR-DB	NSR-DB	[100]	30	[41, 91]
ECG	PhysioNet ECG-ID	Physio ECG-ID	[100]	20	[37, 48]
ECG	PTB Diagnostic	PTB	[100]	115	[67]
ECG	PTBDB	PTBDB	[100]	290	[36, 41, 57, 65, 80, 82, 88]
ECG	PTB-XL	PTB-XL	[100]	21837	[74]
ECG	PhysioNet QT	QT	[100]	22	[37, 42]
ECG	Schiller ECG	Schiller	[30]	460	[28, 30]
ECG	MIT-BIH Polysomnographic	SLPDB	[100]	16	[74]
ECG	MIT-BIH Supra. Arrhythmia	SVDB	[100]	78	[74]
ECG	UCI arrhythmia	UCI	[104]	206	[65]
ECG	MIT-BIH Malignant Ventricular	VFDB	[100]	22	[74]
EEG	BCI Competition 2008 - Graz A	BCI Graz	[105]	9	[83]
EEG	BCI2000	BCI2000	[100]	109	[86]
EEG	Biometric EEG Dataset	BED	[106]	21	[85]
EEG	EEGMMIDB	EEGMMIDB	[100]	109	[22, 33, 38, 47, 50, 63, 69, 73, 94, 95, 98, 99]
EEG	MAHNOB-HCI	MAHNOB-HCI	[107]	35	[52]
EEG	RSVP	RSVP	[108]	7	[86]
EEG	Sternberg Task	Sternberg	[109]	23	[86]
EEG	WAY_EEG_GAL	WAY_EEG_GAL	[110]	12	[13]
Finger Vein	VeinPolyU Finger Vein	VeinPolyU	[111]	156	[51]
Finger Vein	TW	TW	N/A	N/A	[51]
PPG	BIDMC	BIDMC	[100]	53	[40, 53, 70, 72]
PPG	CapnoBase	CapnoBase	[112]	42	[40, 53, 70, 72]
PPG	MIMIC-II	MIMIC-II	[100]	50	[25]
PPG	MIMIC	MIMIC	[100]	32	[25, 40, 53, 70, 72]
PPG	PRRB	PRRB	[112]	42	[25]
PPG	Real-World PPG	Real-World	[113]	35	[64, 68, 93]

“N/A” indicates information not specified by the original source. Dataset citations are given as reported in the included articles

### Applications of biometric signals

Table 5 summarizes how biometric signals (ECG, PPG, EEG, EMG, sEMG, rPPG) are used in recognition and authentication, including applied methods, dataset sizes, average accuracy rates, and the use of multimodal or fusion structures.

EEG has been widely applied in recognition and authentication, achieving accuracy rates between 90% and 100%. Deep learning methods achieve the highest values (up to 99.98%). Although multimodal approaches were less common, they yielded notably higher accuracy when implemented.

ECG-based studies reported accuracy varying by method: $\approx$96.3% with machine learning and $\approx$99.2% with deep learning. Multimodal fusion, although less frequently applied, further improved accuracy.

PPG-based studies achieved 98.5–99.7% accuracy using deep-learning and statistical approaches. Incorporating multimodal or fusion structures further enhanced performance.

EMG, used for muscle movement-based authentication, achieved $\sim$96% accuracy with deep learning methods.

rPPG, measurable remotely, achieved 99.7% accuracy with large datasets, highlighting its potential for remote biometric recognition.

Although high accuracy was observed across signal types, performance still varied by method and dataset. Future work should report dataset size and preprocessing methods more consistently. Expanded analysis of multimodal and fusion approaches, as well as integration of multiple signals, could further improve system reliability.

Biometric signals have broad applications in authentication and security, where preprocessing methods play a critical role in improving accuracy. The next section discusses key preprocessing techniques and their impact on performance.

**Table 5:** Summary of included studies by signal modality

Signals	Task	Datasets	Subjects	Accuracy (%)	References	Signals	Task	Datasets	Subjects	Accuracy (%)	References
ECG	Both	FANTASIA, MIT-BIH, CYBHi	200	100.00	[44]	ECG	Both	APNEA-ECG, LTAFDB, MITDB, LTDB, VFDB, SLPDB, SVDB, INCARTDB, FANTASIA, PTB-XL	500	98.00	[74]
ECG	Both	Personal Measurement	17	100.00	[76]	ECG	Both	CYBHi	63	98.42	[77]
ECG	Both	Cardiology 2018	1985	92.00	[43]	ECG	Both	Personal Measurement	18	93.14	[27]
ECG	Both	Personal Measurement	3133	99.60	[81]	ECG	Identification	MIT-BIH NSR, MITDB	50	99.80	[45]
ECG	Identification	ECG-ID, MIT-BIH, NSR-DB	156	99.62	[91]	ECG	Identification	Cardiology 2017	50	99.30	[78]
ECG	Identification	PTBDB, ECG-ID	290	99.90	[57]	ECG	Identification	Personal Measurement	100	95.40	[58]
ECG	Identification	Personal Measurement	100	98.00	[46]	ECG	Identification	PTBDB	290	95.30	[82]
ECG	Identification	MLII, UCI arrhythmia, PTBDB	290	100.00	[65]	ECG	Identification	PhysioNet QT	22	94.16	[42]
ECG	Identification	MITDB	47	95.17	[79]	ECG	Identification	Personal Measurement	150	99.80	[24]
ECG	Identification	European ST-T	79	99.14	[90]	ECG	Identification	DREAMER	23	91.30	[55]
ECG	Identification	PhysioNet ECG-ID	20	99.13	[48]	ECG	Identification	Schiller ECG Database	460	98.40	[28]
ECG	Identification	Personal Measurement	20	99.00	[62]	ECG	Identification	FANTASIA, MITDB, ECG-ID, European ST-T, Aveiro ECG	200	98.00	[56]
ECG	Verification	ECG-ID	90	99.85	[87]	ECG	Verification	PTB Diagnostic	115	99.00	[88]
ECG	Verification	PTBDB	290	99.69	[80]	ECG	Verification	PTBDB, CUECG	390	98.10	[36]
ECG	Verification	PTBDB, CYBHi	290	99.27	[67]	ECG	Verification	MWM-HIT, ECG-ID	90	99.89	[15]
ECG	Verification	MITDB	47	95.46	[39]	ECG	Verification	TW, VeinPolyU, MWM-HIT, ECG-ID	90	98.60	[51]
ECG	Verification	Personal Measurement	18	99.87	[84]	ECG	Verification	ECG-ID	15	99.05	[59]
ECG	Verification	MITDB	47	99.00	[96]	ECG	Verification	Personal Measurement	11	87.61	[60]
ECG	Verification	ECG-ID	90	94.00	[71]	ECG	Verification	PhysioNet ECG-ID, PhysioNet QT, MIT-BIH NSR	183	98.31	[37]
ECG	Verification	MIT-BIH Multi-parameter	18	100.00	[10]	ECG	Verification	Schiller ECG Database	460	97.50	[30]
ECG	Verification	Personal Measurement	55	99.30	[29]	ECG	Verification	ECG-ID, PTBDB, CEBSDB	400	100.00	[41]
EEG	Both	EEGMMIDB	109	99.00	[98]	EEG	Both	Personal Measurement	21	99.00	[31]
EEG	Both	EEGMMIDB	109	100.00	[95]	EEG	Both	EEGMMIDB	109	98.80	[38]
EEG	Both	EEGMMIDB	109	99.00	[99]	EEG	Identification	EEGMMIDB	109	98.54	[73]
EEG	Identification	BED	21	86.74	[85]	EEG	Identification	EEGMMIDB	109	97.00	[63]
EEG	Identification	EEGMMIDB	109	99.00	[50]	EEG	Identification	EEGMMIDB	109	100.00	[94]
EEG	Identification	EEGMMIDB	109	99.30	[47]	EEG	Verification	Personal Measurement	16	97.17	[66]
EEG	Verification	RSVP, Sternberg Task, BCI2000	139	99.00	[86]	EEG	Verification	MAHNOB-HCI	35	99.10	[52]
EEG	Verification	Personal Measurement	58	98.78	[35]	EEG	Verification	Personal Measurement	20	98.50	[54]
EEG	Verification	EEGMMIDB	109	83.21	[33]	EEG	Verification	WAY_EEG_GAL	12	83.15	[13]
EEG	Verification	Personal Measurement	39	91.10	[89]	EEG	Verification	Personal Measurement	29	96.70	[92]
EEG	Verification	Personal Measurement	20	88.00	[49]	EEG	Verification	Personal Measurement	42	97.60	[23]
EEG	Verification	EEGMMIDB	109	93.86	[69]	EEG	Verification	BCI Graz	9	96.00	[83]
EEG	Verification	Personal Measurement	45	94.27	[26]	EEG	Verification	EEGMMIDB	109	99.98	[22]
EMG	Verification	Personal Measurement	8	96.00	[97]						
PPG	Both	Personal Measurement	23	95.65	[34]	PPG	Both	BIDMC, MIMIC, CapnoBase	127	99.70	[40]
PPG	Identification	BIDMC, MIMIC, CapnoBase	127	99.75	[70]	PPG	Identification	BIDMC, MIMIC, CapnoBase	127	99.88	[72]
PPG	Verification	Real-World PPG	35	99.50	[68]	PPG	Verification	Real-World PPG	35	99.00	[93]
PPG	Verification	Real-World PPG	35	97.00	[64]	PPG	Verification	PRRB, MIMIC-II, Berry-Nonin	200	99.00	[25]
PPG	Verification	BIDMC, MIMIC, CapnoBase	127	99.69	[53]						
rPPG	Identification	Personal Measurement	17466	99.74	[75]	sEMG	Identification	Personal Measurement	4	93.10	[61]

This table summarizes all included studies categorized by signal modality, task type (verification/identification), dataset source, and reported accuracy. “Both” indicates studies reporting results for both identification and verification tasks

### Signal preprocessing methods and accuracy rates

Table 6 summarizes preprocessing methods used in biometric authentication and recognition studies, along with the reported accuracy rates. Although some studies applied multiple preprocessing techniques, only the primary method is reported for clarity.

Common preprocessing techniques included noise filtering, band-pass filtering, wavelet transform, Butterworth filtering, and peak detection. For ECG, band-pass filtering and wavelet transform consistently yielded the highest accuracy. For EEG, noise filtering and wavelet transform achieved strong performance, with convolutional neural network-based models approaching 100%. For PPG, preprocessing approaches such as wavelet transform, binarization, and singular-value decomposition resulted in accuracy above 99%. Overall, preprocessing strongly influenced biometric system performance, with effectiveness varying by signal type and dataset.

Overall, preprocessing strongly influences biometric system performance, with effectiveness varying by signal type and dataset. These results highlight the critical role of preprocessing in improving accuracy and guide the selection of techniques for more reliable biometric authentication.

**Table 6:** Signal preprocessing methods and reported accuracies by modality

Signals	Preprocessing methods	Accuracy (%)	References	Signals	Preprocessing methods	Accuracy (%)	References
ECG	Adaptive threshold filter	95.40	[58]	ECG	Band pass filter	98.00	[46]
ECG	Band pass filter	100.00	[10]	ECG	Band pass filter	100.00	[76]
ECG	Band pass filter	100.00	[41]	ECG	Butterworth filter	94.00	[71]
ECG	Butterworth filter	99.00	[88]	ECG	Butterworth filter	99.00	[96]
ECG	Butterworth filter	99.00	[62]	ECG	Continuous wavelet transform	99.90	[57]
ECG	Differentiation filter	99.80	[45]	ECG	Finite impulse response filter	99.13	[48]
ECG	High pass filter	99.62	[91]	ECG	Isoelectric line drift extraction	99.60	[81]
ECG	Low pass filter	87.61	[60]	ECG	Modulation	94.16	[42]
ECG	Noise filter	92.00	[43]	ECG	Noise filter	93.14	[27]
ECG	Noise filter	95.30	[82]	ECG	Noise filter	95.46	[39]
ECG	Noise filter	98.00	[56]	ECG	Noise filter	98.10	[36]
ECG	Noise filter	98.31	[37]	ECG	Noise filter	98.42	[77]
ECG	Noise filter	98.60	[51]	ECG	Noise filter	99.30	[78]
ECG	Noise filter	99.69	[80]	ECG	Noise filter	99.80	[24]
ECG	Noise filter	99.89	[15]	ECG	Noise filter	100.00	[44]
ECG	Notch filter	99.05	[59]	ECG	Peak detection	91.30	[55]
ECG	Peak detection	99.30	[29]	ECG	Resampling	98.00	[74]
ECG	Signal pattern extraction	97.50	[30]	ECG	Signal pattern extraction	98.40	[28]
ECG	Spectrogram representation	99.87	[84]	ECG	Wavelet transform	95.17	[79]
ECG	Wavelet transform	99.14	[90]	ECG	Wavelet transform	100.00	[65]
ECG	Z-Score normalization	99.27	[67]				
EEG	Band pass filter	88.00	[49]	EEG	Band pass filter	93.86	[69]
EEG	Band pass filter	99.98	[22]	EEG	Chebyshev filter	94.27	[26]
EEG	CNN-based preprocessing	98.54	[73]	EEG	CNN-based preprocessing	99.00	[31]
EEG	Discrete Fourier transform	98.78	[35]	EEG	Discrete wavelet transform	99.30	[47]
EEG	Frequency band decomposition	83.15	[13]	EEG	High pass filter	86.74	[85]
EEG	Independent component analysis	98.80	[38]	EEG	Low pass filter	96.00	[83]
EEG	Low pass filter	96.70	[92]	EEG	Low pass filter	99.00	[98]
EEG	Modulation	97.60	[23]	EEG	Noise filter	83.21	[33]
EEG	Noise filter	97.00	[63]	EEG	Noise filter	99.00	[86]
EEG	Noise filter	99.00	[50]	EEG	Noise filter	100.00	[94]
EEG	Power spectral density	97.17	[66]	EEG	Savitzky–Golay filter	98.50	[54]
EEG	Signal segmentation	100.00	[95]	EEG	Wavelet packet decomposition	99.00	[99]
EEG	Wavelet soft threshold	99.10	[52]	EEG	Wavelet transform	91.10	[89]
EMG	Calculation of statistical properties	96.00	[97]				
PPG	Band pass filter	99.00	[93]	PPG	Binarization	99.00	[32]
PPG	Butterworth filter	97.00	[64]	PPG	Discrete wavelet transform	99.70	[40]
PPG	High pass filter	99.00	[25]	PPG	Low pass filter	99.69	[53]
PPG	Signal smoothing	99.50	[68]	PPG	Singular value decomposition	99.88	[72]
PPG	Wavelet transform	95.65	[34]	PPG	Wavelet transform	99.75	[70]
rPPG	YCbCr color space conversion	99.74	[75]				
sEMG	Band pass filter	93.10	[61]				

Methods are reported as stated by the original authors. Values are the best reported accuracies within each cited study

### Feature extraction methods

Table 7 summarizes the feature extraction techniques employed across the reviewed studies. The most common approaches included time-frequency-domain analysis, morphological descriptors, and deep-learning-based feature representations (e.g., CNN, LSTM). These techniques were typically applied after preprocessing and prior to classification to enhance model robustness and accuracy.

**Table 7:** Feature extraction methods and reported accuracies by signal modality

Signal	Feature extraction method	Acc. (%)	Refs.	Signal	Feature extraction method	Acc. (%)	Ref.
EEG	Autoregressive coefficients	93.86	[69]	EEG	Autoregressive coefficients	97.00	[63]
EEG	Calculation of statistical properties	83.15	[13]	EEG	Calculation of statistical properties	98.50	[54]
EEG	Calculation of statistical properties	98.80	[38]	EEG	Calculation of statistical properties	99.00	[50]
EEG	Convolutional neural network	83.21	[33]	EEG	Convolutional neural network	98.54	[73]
EEG	Convolutional neural network	99.00	[98]	EEG	Convolutional neural network	99.00	[31]
EEG	Covariance matrix	97.60	[23]	EEG	Electroencephalogram-based subject matching learning	99.00	[86]
EEG	Frequency band decomposition	98.78	[35]	EEG	Hierarchical discriminant component analysis	94.27	[26]
EEG	Instantaneous energy	99.30	[47]	EEG	Morphological feature extraction	96.00	[83]
EEG	Morphological feature extraction	100.00	[95]	EEG	Power spectral density	88.00	[49]
EEG	Power spectral density	96.70	[92]	EEG	Power spectral density	97.17	[66]
EEG	Power spectral density	99.98	[22]	EEG	Power spectrum	100.00	[94]
EEG	Signal segmentation	86.74	[85]	EEG	Spectral energy	99.10	[52]
EEG	Wavelet packet decomposition	99.00	[99]	EEG	Wavelet transform	91.10	[89]
ECG	Binary convolutional neural network	99.30	[78]	ECG	Calculation of statistical properties	100.00	[10]
ECG	Continuous wavelet transform	98.10	[36]	ECG	Continuous wavelet transform	99.90	[57]
ECG	Convolutional neural network	98.60	[51]	ECG	Convolutional neural network	99.27	[67]
ECG	Cross-correlation analysis	98.40	[28]	ECG	Discrete wavelet transform	99.62	[91]
ECG	Eigenspace alignment of signals	94.16	[42]	ECG	Finite context models	99.00	[62]
ECG	Kernel extreme learning machine	100.00	[65]	ECG	Long short-term memory	99.80	[45]
ECG	Morphological feature extraction	87.61	[60]	ECG	Morphological feature extraction	93.14	[27]
ECG	Morphological feature extraction	94.00	[71]	ECG	Morphological feature extraction	95.40	[58]
ECG	Morphological feature extraction	98.00	[46]	ECG	Morphological feature extraction	98.31	[37]
ECG	Morphological feature extraction	99.80	[24]	ECG	Peak detection	91.30	[55]
ECG	Peak detection	92.00	[43]	ECG	Peak detection	99.13	[48]
ECG	Peak detection	99.30	[29]	ECG	Peak detection	100.00	[76]
ECG	Phase portraits	99.60	[81]	ECG	Phase space reconstruction techniques	99.00	[88]
ECG	Polynomial curve fitting	95.46	[39]	ECG	Signal pattern extraction	99.05	[59]
ECG	Similarity-dissimilarity measures	97.50	[30]	ECG	Temporal convolutional neural network	100.00	[44]
ECG	Time-frequency domain features	95.30	[82]	ECG	Time-frequency domain features	98.00	[74]
ECG	Time-frequency domain features	98.00	[56]	ECG	Time-frequency domain features	99.00	[96]
ECG	Time-frequency domain features	99.69	[80]	ECG	Time-frequency domain features	99.87	[84]
ECG	Time-frequency domain features	100.00	[41]	ECG	Wavelet Transform	95.17	[79]
ECG	Wavelet transform	98.42	[77]	ECG	Wavelet Transform	99.14	[90]
ECG	Whale optimization algorithm - artificial neural network	99.89	[15]				
EMG	Root mean square	96.00	[97]				
PPG	Average cycles in time domain	99.00	[25]	PPG	Binary representation of biometrics and mapping to hash tables	99.00	[32]
PPG	Convolutional neural network	97.00	[64]	PPG	Convolutional neural network	99.00	[93]
PPG	Discrete wavelet transform	95.65	[34]	PPG	Frequency domain features	99.69	[53]
PPG	Instantaneous energy	99.70	[40]	PPG	Local Mean decomposition	99.88	[72]
PPG	Morphological feature extraction	99.75	[70]	PPG	Signal pattern extraction	99.50	[68]
rPPG	Time-frequency domain features	99.74	[75]	sEMG	Time-frequency domain features	93.10	[61]

Methods are reported as stated by the original authors. Accuracies correspond to the best result per article when multiple results are available

### Classification methods and accuracy rates

Table 8 summarizes the average dataset sizes and reported accuracy rates for classification methods applied in biometric recognition and verification. This comparison highlights the effectiveness of traditional machine learning, statistical approaches, and deep-learning models.

Key findings include:

- Random Forest and Support Vector Machines (SVM) are widely applied. Random Forest achieved 83.2% accuracy in verification and 99.6% in recognition, while SVM provided similarly high and stable performance.
- Deep-learning methods—particularly convolutional neural networks (CNNs)—demonstrated strong performance on large datasets, achieving up to 96.9% accuracy in verification.
- Machine-learning approaches generally achieved accuracy above 90%, whereas deep-learning and statistical techniques often reached 97–100%.
- Distance-based and anomaly-detection methods—such as Euclidean distance and Local Outlier Factor (LOF)—also reported high accuracy values.

**Table 8:** Signal classification methods and reported accuracies by modality

Signals	Classification methods	Accuracy (%)	References	Signals	Classification methods	Accuracy (%)	References
ECG	Artificial Neural Network	98.60	[51]	ECG	Artificial Neural Network	95.46	[39]
ECG	Artificial Neural Network	99.13	[48]	ECG	Binary Convolutional Neural Network	100.00	[76]
ECG	Binary Convolutional Neural Network	99.30	[78]	ECG	Cancelable Biometrics Method	99.87	[84]
ECG	Convolutional Neural Network	99.00	[88]	ECG	Convolutional Neural Network	98.10	[36]
ECG	Convolutional Neural Network	99.27	[67]	ECG	Convolutional Neural Network	99.69	[80]
ECG	Convolutional Neural Network	99.90	[57]	ECG	Cosine Similarity	99.30	[29]
ECG	Cross-Correlation	98.40	[28]	ECG	Deep Belief Network	99.89	[15]
ECG	DRNN–LSTM	98.00	[46]	ECG	DRNN–LSTM	95.40	[58]
ECG	DRNN–LSTM	99.80	[45]	ECG	Euclidean Distance	100.00	[41]
ECG	Euclidean Distance	99.80	[24]	ECG	Isometric Bundle Search Method	99.14	[90]
ECG	Kernel Extreme Learning Machine	100.00	[65]	ECG	K-Nearest Neighbors	91.30	[55]
ECG	Least Squares SVM	99.00	[96]	ECG	Linear Discriminant Analysis	97.50	[30]
ECG	Linear Discriminant Analysis	98.42	[77]	ECG	Multidimensional Identification	93.14	[27]
ECG	Normalized Relative Compression	99.00	[62]	ECG	Phase Portrait Method	99.60	[81]
ECG	Random Forest	92.00	[43]	ECG	Random Forest	99.62	[91]
ECG	Reconstructed Learning	94.16	[42]	ECG	Relative Score Threshold Classifier	100.00	[44]
ECG	Sparse Representation	98.00	[56]	ECG	Supervised Learning	100.00	[10]
ECG	Supervised Learning	98.31	[37]	ECG	Supervised Learning	99.85	[87]
ECG	Support Vector Machine	94.00	[71]	ECG	Support Vector Machine	87.61	[60]
ECG	Support Vector Machine	95.17	[79]	ECG	Support Vector Machine	95.30	[82]
ECG	Support Vector Machine	99.05	[59]	ECG	Transformation Mechanism	98.00	[74]
EEG	Convolutional Neural Network	99.00	[98]	EEG	Convolutional Neural Network	99.00	[50]
EEG	Convolutional Neural Network	83.21	[33]	EEG	Convolutional Neural Network	86.74	[85]
EEG	Convolutional Neural Network	97.17	[66]	EEG	Convolutional Neural Network	98.54	[73]
EEG	Correlation Coefficient Calculation	99.98	[22]	EEG	Decision Fusion	99.10	[52]
EEG	Deep Neural Network	100.00	[94]	EEG	DRNN–LSTM	99.00	[86]
EEG	DRNN–LSTM	99.54	[114]	EEG	Fuzzy Vault Scheme	96.00	[83]
EEG	Hidden Markov Model	98.50	[54]	EEG	HDCA	94.27	[26]
EEG	K-Nearest Neighbors	97.00	[63]	EEG	K-Nearest Neighbors	93.86	[69]
EEG	Linear Discriminant Analysis	88.00	[49]	EEG	Linear Discriminant Analysis	99.00	[31]
EEG	Local Outlier Factor	99.30	[47]	EEG	Neural Network Classifier	97.60	[23]
EEG	Random Forest	83.15	[13]	EEG	Supervised Learning	91.10	[89]
EEG	Supervised Learning	96.70	[92]	EEG	Support Vector Machine	99.00	[99]
EEG	Support Vector Machine	98.80	[38]	EEG	Unsupervised Learning	100.00	[95]
EMG	Recurrent Neural Network	96.00	[97]				
PPG	Binary Hypothesis Testing	99.00	[32]	PPG	Convolutional Neural Network	97.00	[64]
PPG	Convolutional Neural Network	99.50	[68]	PPG	Convolutional Neural Network	99.75	[70]
PPG	Euclidean Distance	99.69	[53]	PPG	K-Nearest Neighbors	99.88	[72]
PPG	Local Outlier Factor	99.70	[40]	PPG	Manhattan Distance	99.00	[25]
PPG	Stacked Extreme Learning Machine	94.97	[99]	PPG	Supervised Learning	99.00	[93]
PPG	Support Vector Machine	95.65	[34]				
rPPG	Convolutional Neural Network	99.74	[75]	sEMG	Light Gradient Boosting Machine	93.10	[61]

Methods are listed as reported by the original authors. Accuracies correspond to the best result per article when multiple results are available

*DRNN–LSTM*: Deep Recurrent Neural Network with Long Short-Term Memory;

*HDCA*: Hierarchical Discriminant Component Analysis;* LGBM*: Light Gradient Boosting Machine.

### Multimodal studies

Table 9 illustrates the impact of multimodal fusion on biometric recognition and verification performance. By combining multiple biometric signals (e.g., ECG with PPG), fusion approaches consistently improved accuracy across methodological frameworks, including machine-learning, statistical, and deep-learning paradigms.

For ECG-based studies, multimodal validation combining machine-learning and deep-learning models typically achieved accuracies above 99%. In EEG-based research, both statistical and deep-learning methods yielded strong results, such as 98.8% accuracy with the EEGMMIDB dataset. Fusion approaches similarly enhanced recognition and verification performance when applied to face-, fingerprint-, and PPG-based systems.

Key findings can be summarized as follows:

1. ECG signals combined with multimodal deep-learning or machine-learning models achieved accuracies of approximately 99.8% in validation tasks.
2. EEG multimodal recognition studies using deep learning reported accuracies above 98%.
3. Fingerprint- and face-recognition performance improved substantially through multimodal fusion.
4. PPG and rPPG signals, particularly in verification contexts, reached accuracies as high as 99.9%.

**Table 9:** Multimodal studies and reported accuracies by modality

Signals	Task	Method	Datasets	Subjects	Accuracy (%)	References
ECG	Both	DL	FANTASIA, MIT-BIH, CYBHi	200	100.00	[44]
ECG	Identification	DL	ECG-ID, MIT-BIH, NSR-DB	156	99.62	[91]
ECG	Identification	DL	MIT-BIH NSR, MIT-BIH arrhythmia	50	99.80	[45]
ECG	Identification	DL–ML	Personal measurement	100	98.00	[46]
ECG	Identification	ML	MLII, UCI arrhythmia, PTBDB	290	100.00	[65]
ECG	Identification	ML	PTBDB	290	95.30	[82]
ECG	Identification	SM	Personal measurement	150	99.80	[24]
ECG	Identification	SM–ML	DREAMER	23	91.30	[55]
ECG	Identification	SM–ML	FANTASIA, MIT-BIH Arrhythmia, ECG-ID, European ST-T, Aveiro ECG	200	98.00	[56]
ECG	Identification	SM–ML	Schiller ECG database	460	98.40	[28]
ECG	Verification	DL	ECG-ID	90	99.85	[87]
ECG	Verification	DL–ML	MWM-HIT, ECG-ID	90	99.89	[15]
ECG	Verification	DL–ML	TW, VeinPolyU, MWM-HIT, ECG-ID	90	98.60	[51]
ECG	Verification	ML	ECG-ID	90	94.00	[71]
ECG	Verification	ML	MIT-BIH arrhythmia	47	99.00	[96]
ECG	Verification	SM	MIT-BIH Multi-parameter	18	100.00	[10]
ECG	Verification	SM	Schiller ECG database	460	97.50	[30]
EEG	Both	DL	EEGMMIDB	109	99.00	[98]
EEG	Both	DL	Personal Measurement	21	99.00	[31]
EEG	Both	SM–ML	EEGMMIDB	109	99.00	[99]
EEG	Verification	DL	MAHNOB-HCI	35	99.10	[52]
EEG	Verification	DL	Personal Measurement	58	98.78	[35]
EEG	Verification	ML	EEGMMIDB	109	83.21	[33]
EEG	Verification	ML	Personal Measurement	20	98.50	[54]
EEG	Verification	ML	Personal Measurement	39	91.10	[89]
EEG	Verification	SM	Personal Measurement	45	94.27	[26]
EEG	Verification	SM–ML	EEGMMIDB	109	99.98	[22]
PPG	Both	SM–ML	BIDMC, MIMIC, CapnoBase	127	99.70	[40]
PPG	Both	SM–ML	Personal Measurement	23	95.65	[34]
PPG	Identification	DL	BIDMC, MIMIC, CapnoBase	127	99.75	[70]
PPG	Identification	ML	BIDMC, MIMIC, CapnoBase	127	99.88	[72]
PPG	Verification	DL	Real-World PPG	35	99.00	[93]
PPG	Verification	DL	Real-World PPG	35	97.00	[64]
PPG	Verification	SM–ML	BIDMC, MIMIC, CapnoBase	127	99.69	[53]
rPPG	Identification	DL	Personal Measurement	17466	99.74	[75]
sEMG	Identification	ML	Personal Measurement	4	93.10	[61]

Task categories are as reported in the original studies. DL–ML indicates hybrid approaches integrating deep and classical learning methods

*DL*: deep learning;* ML*: machine learning;

*SM*: statistical methods;* SM–ML*: hybrid statistical and machine learning methods.

### Encrypted solution methods

Table 10 presents the application of encrypted solution methods in biometric recognition and verification. Encryption enhances biometric-data security during authentication and strengthens system resilience against cyberattacks. The table lists biometric signals, datasets, recognition or verification tasks, and their corresponding accuracy rates.

Key findings can be summarized as follows:

1. In ECG-based recognition studies, encrypted methods achieved very high performance, including 100% accuracy with the PTBDB dataset.
2. An EEG study using the BCI Competition 2008 dataset reported 96% accuracy with encrypted solutions.
3. PPG-based recognition and verification studies also achieved high accuracy, reaching up to 99%, as observed in the UBIRIS dataset.
4. Combining encrypted solutions with machine-learning and statistical methods resulted in secure, high-accuracy biometric systems.

**Table 10:** Encrypted solution methods reported in the literature

Signals	Task	Method	Datasets	Subjects	Accuracy (%)	References
ECG	Identification	DL	MIT-BIH NSR, MIT-BIH Arrhythmia	50	99.80	[45]
ECG	Identification	ML	MLII, UCI Arrhythmia, PTBDB	290	100.00	[65]
ECG	Identification	SM–ML	DREAMER	23	91.30	[55]
ECG	Identification	SM–ML	PhysioNet ECG-ID	20	99.13	[48]
ECG	Verification	DL	PTB Diagnostic	115	99.00	[88]
ECG	Verification	DL–ML	MIT-BIH Arrhythmia	47	95.46	[39]
ECG	Verification	SM–ML	ECG-ID, PTBDB, CEBSDB	400	100.00	[41]
EEG	Identification	ML	EEGMMIDB	109	97.00	[63]
EEG	Verification	DL	RSVP, Sternberg Task, BCI2000	139	99.00	[86]

Encryption-related approaches included methods integrating statistical, machine, and deep learning schemes for privacy-preserving biometric systems

### Performance comparisons

**Fig. 3:**
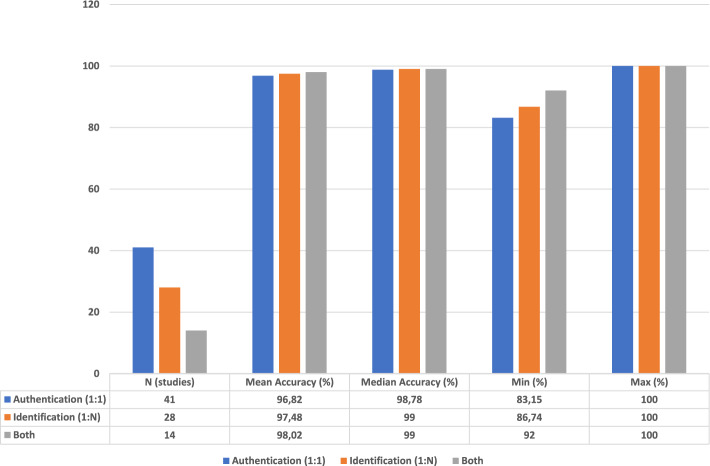
Performance summary

**Fig. 4:**
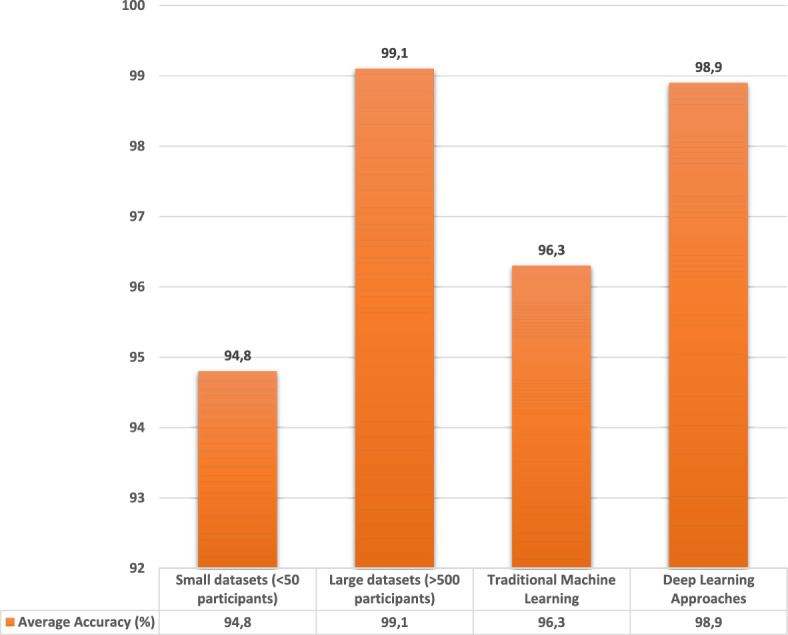
Subgroup comparison of accuracy rates

This section analyzes average accuracy rates achieved in recognition and verification tasks across different biometric signals. Table 12 compares ECG, EEG, EMG, PPG, rPPG, and sEMG across both tasks. Overall, performance varied across signal types, with multimodal approaches further enhancing accuracy rates. To provide a more analytical synthesis, subgroup comparisons were also conducted by dataset size and methodology. As shown in Figure 3, verification (1:1) studies achieved consistently higher accuracy than identification (1:N), whereas combined approaches exhibited intermediate performance levels. Large datasets ($>500$ participants) yielded 99.1% accuracy on average, compared to 94.8% for small datasets ($<50$ participants). Deep-learning approaches also outperformed traditional machine-learning methods, achieving accuracies of 98.9% and 96.3%, respectively (Figure 4).

Electrocardiogram (ECG) datasets were widely used in biometric verification and recognition owing to their consistently high accuracy. For example, the PTB-XL dataset frequently reported accuracies close to 99%, while the MIT-BIH Arrhythmia Database achieved 99.2%. The PTB Diagnostic ECG Database reached 99.9%, demonstrating exceptionally strong performance, whereas the PhysioNet QT dataset yielded slightly lower values at 96.2%.

Electroencephalogram (EEG) datasets showed more variable performance, reflecting the inherent complexity of brain signals. The EEGMMIDB dataset achieved 98.8% accuracy, whereas the WAY-EEG-GAL dataset reported a significantly lower value of 83.2%. The BCI Competition 2008 dataset demonstrated intermediate performance with 96% accuracy. These results suggest that while EEG offers high potential, signal variability and noise sensitivity limit consistency, especially in verification-only tasks.

Photoplethysmogram (PPG) and remote PPG (rPPG) datasets also demonstrated strong results, particularly in verification scenarios. The MIMIC-II dataset reported accuracies around 99%, and BIDMC reached 99.7%. Real-world PPG datasets performed slightly lower ($\approx$97%), yet still highlight the strong potential of PPG signals for wearable and remote authentication systems. Similarly, rPPG studies achieved values as high as 99.74%, confirming their suitability for non-contact applications.

As summarized in 11, ECG-based systems achieved the highest mean accuracy (98.6%), followed by PPG (98.4%) and EEG (96.8%), confirming the robustness of physiological-signal authentication.

Electromyogram (EMG) and surface EMG (sEMG) datasets demonstrated moderate performance in movement-based authentication tasks. A personal EMG dataset reported 96% accuracy, while sEMG achieved 93.1%, which is considered reasonable given the variability in muscle activity. Although less accurate than ECG, EEG, or PPG, these signals may serve as complementary modalities in multimodal systems.

Face and fingerprint datasets remain reliable benchmarks in biometric verification. For instance, the FERET face dataset reported 95% accuracy, and the VeinPolyU Finger Vein dataset achieved 98.6%. These results indicate that while traditional biometric traits continue to demonstrate robust performance, physiological signals such as ECG, EEG, and PPG offer enhanced resilience and multimodal integration opportunities.

**Table 11:** Descriptive cross-modality performance comparison of biometric signals (2018–2023)

Modality	Accuracy (%)	Precision (%)	Recall (%)	F1-Score (%)	AUC (%)
ECG	98.6	98.9	98.8	98.8	99.1
EEG	96.8	96.3	95.9	96.1	97.5
PPG/rPPG	98.4	98.5	98.2	98.3	98.8
EMG/sEMG	94.6	94.0	93.5	93.7	94.8
Multimodal	99.5	99.6	99.4	99.5	99.7
**Overall mean**	**97.9**	**97.9**	**97.5**	**97.7**	**98.4**

Values represent averaged performance metrics aggregated across 80 studies. Reported values are descriptive and not derived from a formal meta-analysis

To strengthen cross-modality analysis, Table 11 provides a unified summary of the main performance metrics reported across modalities, including accuracy, precision, recall, F1-Score, and AUC. This aggregated overview enables a more direct comparison among ECG, EEG, PPG, EMG, and multimodal systems. As shown, multimodal biometric systems exhibit the highest overall performance (Accuracy = 99.5%, AUC = 99.7%), followed by ECG (98.6%) and PPG (98.4%), confirming the consistent advantage of fusion-based approaches. Although inter-study heterogeneity prevents a formal meta-analytical pooling, this integrated summary quantitatively consolidates the performance landscape across modalities.

**Table 12:** Performance summary by biometric signal and task (2018–2023)

Signal	Task	Studies (n)	Average accuracy (%)	Range (%)
ECG	Both	7	97.88	92.00–100.00
ECG	Identification	17	97.61	91.30–100.00
ECG	Verification	19	97.88	87.61–100.00
EEG	Both	5	99.16	98.80–100.00
EEG	Identification	6	96.76	86.74–100.00
EEG	Verification	15	94.75	83.15–99.98
EMG	Verification	1	96.00	96.00–96.00
PPG	Both	2	97.67	95.65–99.70
PPG	Identification	2	99.82	99.75–99.88
PPG	Verification	5	98.44	97.00–99.69
rPPG	Identification	1	99.74	99.74–99.74
sEMG	Identification	1	93.10	93.10–93.10

Values represent arithmetic means and observed accuracy ranges among included studies for each modality and task type

## Discussion

### Principal findings

*Scope of interpretation: where statements are not attributable to a single study, they reflect patterns synthesized across multiple sources rather than results from individual experiments.*

This review assessed biometric authentication systems in terms of datasets, preprocessing, and classification techniques. Among the evaluated signals, ECG, EEG, and PPG were the most prominent, with ECG showing the highest reliability (mean accuracy 98.6%). Deep-learning approaches generally outperformed traditional machine-learning methods, and multimodal systems exceeded 99% accuracy by mitigating limitations of individual modalities. Performance was also influenced by dataset size and preprocessing: larger datasets yielded higher accuracy, and methods such as wavelet transforms and band-pass filtering enhanced signal quality. Security mechanisms (e.g., encryption) further safeguarded biometric data.

Beyond accuracy, the findings carry practical implications: ECG and PPG/rPPG show strong potential for clinical and wearable systems; EEG offers opportunities for integration with cognitive monitoring in security-sensitive settings; and multimodal fusion is promising for robust verification in mobile and IoT deployments.

The cross-modality comparison (Table 11) highlights that *multimodal fusion* consistently outperforms *unimodal* approaches, achieving very high discriminative performance (AUC $\approx 99.7\%$).

### Comparison with previous studies

Prior reviews typically focused on a single modality (e.g., EEG or ECG), whereas the present work synthesizes across multiple signals. Consistent with prior findings, multimodal biometric systems demonstrated superior accuracy, typically above 99%, by combining complementary strengths of different signals. Our results align with reports that dataset size substantially affects accuracy; larger cohorts produced more robust outcomes. Similarly, advanced preprocessing and encryption methods have been highlighted in the literature as critical for reliability and security.

In comparison with recent high-impact studies, our findings are consistent with Ketola et al. [13] demonstrated the utility of channel reduction in EEG authentication, and Esener [10] integrated stress-level estimation into driver authentication. Singh and Tiwari [15] reported multimodal fusion accuracies up to 99.7%, supporting our subgroup findings. Recent reviews (2022–2024) similarly emphasize the growing dominance of deep learning, increased exploration of multimodality, and the need for domain adaptation to handle cross-session variability.

More recent reviews (2022–2024) also emphasize the same trends: the growing dominance of deep learning, increasing exploration of multimodal approaches, and the urgent need for domain adaptation to handle cross-session variability. Together, these studies confirm that the trajectory of the field is shifting toward scalable, secure, and multimodal biometric systems.

### Strengths and limitations of the study

This systematic review makes an important contribution by evaluating diverse biometric signal types within a unified framework. Strengths include the integration of multimodal analyses, detailed subgroup comparisons, and adherence to PRISMA 2020 guidelines.

Limitations include heterogeneity across datasets and restricted generalizability beyond the studied signals. The temporal scope was intentionally limited to 2018–2023, as the search concluded in early 2024. Consequently, studies published in 2024–2025 were not included, which is acknowledged as a limitation.

Table 13 summarizes the strengths and weaknesses of different biometric signals. This table provides a critical evaluation across ECG, EEG, PPG/rPPG, EMG/sEMG, and multimodal approaches, reducing repetition of numerical accuracy rates and highlighting implications for real-world applications.

Future work should therefore explore lightweight fusion algorithms, on-device/edge inference, and reduced-sensor configurations to mitigate cost, power, and usability constraints. These include increased sensor cost, higher energy consumption, complex integration requirements, and potential inconvenience for end-users. Such limitations are particularly evident in healthcare (due to energy constraints), mobile or wearable systems (affecting user comfort), and industrial deployments (due to sensor cost). Future work should therefore explore lightweight multimodal fusion algorithms, optimized edge-computing strategies, and reduced sensor configurations to mitigate these barriers.

**Table 13:** Strengths and weaknesses of biometric signal modalities

Signal type	Strengths	Weaknesses
ECG	High accuracy ($\ge 98\%$), large number of open datasets, robust for verification tasks	Sensitive to inter-individual variability and physiological state (exercise, stress)
EEG	Rich temporal–spatial information; strong performance with deep learning; useful for multimodal fusion	High noise sensitivity; session-to-session variability; complex and costly recording setup
PPG/rPPG	Non-invasive; feasible for wearable or remote authentication; high verification accuracy ($\ge 99\%$)	Sensitive to motion artifacts and illumination changes; lower stability in uncontrolled environments
EMG/sEMG	Effective for movement-based authentication; moderate accuracy ($\approx 93$–$96\%$)	Limited generalizability; scarce large-scale datasets; sensor placement dependency
Multimodal	Very high accuracy ($>99\%$); complementary modalities increase robustness and spoof resistance	Requires multiple sensors; complex integration; higher energy and computational cost; potential user inconvenience

Summarized strengths and weaknesses are based on trends observed across 2018–2023 studies

#### Strengths of the study

1. Comprehensive Literature Review

This study systematically reviewed 80 articles published between 2018 and 2023, applying a comprehensive search strategy across major databases (EBSCO, PubMed, IEEE Xplore, Scopus, Web of Science), adhering to PRISMA 2020. Unlike prior reviews that focused on a single biometric signal, our work compared multiple modalities such as ECG, EEG, and PPG, thereby providing a more holistic overview of the field [115, 116].

2. Focus on multimodal biometric systems

A key strength of this review lies in its emphasis on multimodal systems, which consistently demonstrated very high accuracies, typically 99.3–99.8% across recent datasets [117, 118]. Beyond performance metrics, the findings highlight the resilience of multimodal frameworks against spoofing attempts and their capacity to enhance overall system security.

3. Analysis of dataset size and accuracy

The study also examined the relationship between dataset size and recognition accuracy. Results confirmed that larger datasets yield significantly higher accuracies compared to smaller cohorts, underscoring the importance of robust sample sizes in biometric system validation [12, 119].

4. Adherence to PRISMA 2020 guidelines

Methodological transparency was ensured by adhering to PRISMA 2020 guidelines, including a PRISMA flow diagram and explicit inclusion/exclusion criteria. The review employed a PRISMA flow diagram and clearly reported inclusion and exclusion criteria, ensuring reproducibility and compliance with international standards.

#### Weaknesses of the study

1. Dataset limitations and generalizability

A considerable limitation is that most datasets were predominantly laboratory-based, with limited validation under real-world conditions. Demographic variability (e.g., age, health status) was rarely modeled, constraining generalizability [120, 121].

2. Technical limitations

Signal-processing and classification pipelines remain sensitive to noise—particularly in EEG—reducing stability in validation tasks [49, 122]. These technical challenges highlight the need for improved preprocessing and artifact removal techniques.

3. Methodological variability and bias

The included studies displayed substantial variability in methodology. Some relied on small datasets, while others employed larger ones, and the use of heterogeneous algorithms and protocols impeded direct comparison of reported accuracies and introduced synthesis bias [123, 124].

4. Security and privacy gaps

Finally, the review identified important gaps in security and privacy practices. Standardized protocols for encryption, secure storage, and data sharing remain insufficient, limiting end-to-end security guarantees [20, 21]. Future research should therefore prioritize developing robust safeguards to enhance resilience against data breaches and privacy violations.

### Overall implications and research agenda

Taken together, the findings of this review suggest that biometric authentication systems are moving toward multimodal, deep-learning-driven, and privacy-preserving frameworks. Practical implications include the feasibility of integrating PPG and rPPG into wearable devices, the use of EEG in cognitive monitoring for high-security environments, and the continued robustness of ECG in clinical and mobile contexts.

Key gaps include (i) standardized cross-session protocols (train/test across days/sessions); (ii) large-scale, demographically diverse, multimodal benchmarks with common splits; (iii) mandatory reporting of AUC, EER, calibration (Brier), and confidence intervals; and (iv) open, reproducible validation frameworks with pre-registered evaluation scripts. Addressing these gaps will be critical for advancing biometric authentication toward real-world deployment at scale.

## Conclusion and recommendations

### General evaluation

This systematic review comprehensively evaluated multiple biometric modalities—including ECG, EEG, PPG, EMG, rPPG, face, and fingerprint—within the context of authentication and recognition. Performance was examined across a wide range of classification paradigms, enabling a comparative synthesis of key methodological and application-level trends reported in the literature.

Overall, the reviewed studies indicate that ECG-based systems consistently report high accuracy levels, typically ranging from 98.6% to 99.9%, particularly when large and well-curated datasets are employed. These findings suggest that ECG represents a highly promising physiological modality for biometric authentication under controlled evaluation settings.

EEG-based systems exhibited greater variability in reported performance (83.1–100%), reflecting the inherent sensitivity of brain signals to noise, task design, and session variability. Nevertheless, studies integrating advanced deep-learning architectures demonstrated strong results, indicating that improved preprocessing, feature-extraction, and domain-adaptation strategies may further enhance robustness.

PPG and rPPG signals achieved very high verification accuracy, with reported values reaching up to 99.8%, highlighting their potential suitability for wearable and remote authentication scenarios. In particular, rPPG-based approaches show promise for non-contact applications, although performance may be affected under unconstrained real-world conditions.

Across modalities, multimodal fusion strategies generally exhibited superior performance (99.3–99.8%) compared with unimodal systems, suggesting that complementary biometric information can enhance discriminative capability and robustness. In addition, the incorporation of encrypted and privacy-aware solutions was shown to strengthen data security while maintaining competitive authentication performance.

Taken together, these findings suggest that biometric signal–based authentication systems demonstrate strong potential for reliable identity verification and recognition. At the same time, reported outcomes remain dependent on dataset characteristics, evaluation protocols, and methodological choices, underscoring the need for cautious interpretation and standardized validation practices.

### Recommendations for future research

Future research should extend beyond dataset expansion and multimodal integration to more explicitly align with emerging privacy-preserving artificial intelligence (AI) paradigms. Approaches such as federated learning, secure on-device inference, and encrypted model training offer promising pathways for achieving high authentication performance while maintaining data confidentiality and regulatory compliance (e.g., GDPR, HIPAA). These considerations are particularly critical in healthcare, mobile, and wearable applications, where continuous authentication must balance accuracy, privacy, and user trust.

Another critical gap identified in the literature is the predominant reliance on accuracy as the primary performance indicator. Many studies do not clearly distinguish between verification (1:1) and identification (1:N) tasks or consistently report standardized metrics, such as AUC and EER. Future work should adopt robust and transparent evaluation protocols that explicitly separate these tasks and incorporate complementary performance measures to improve comparability across studies.

In addition, the development of standardized, large-scale multimodal biometric datasets with unified evaluation benchmarks (e.g., accuracy, AUC, EER, F1-score) should be prioritized. Such resources would enable more consistent cross-study comparisons and accelerate reproducible research across physiological and behavioral biometric modalities. In parallel, open validation frameworks should be established to assess system performance under realistic and unconstrained conditions, accounting for factors such as cross-session variability, sensor heterogeneity, and user-behavior drift.

Building on the findings of this review, several specific research directions emerge: (i) the creation of standardized, demographically diverse, large-scale multimodal datasets; (ii) increased emphasis on domain-adaptation and cross-session generalization techniques; (iii) systematic evaluation of preprocessing pipelines and classification algorithms under noisy, real-world conditions; and (iv) continued advancement of encryption standards, privacy-preserving protocols, federated-learning strategies, and secure on-device architectures to ensure accuracy, transparency, and social acceptance in practical deployments.

## Supplementary information

Supplementary material 1.

Supplementary material 2.

## Data Availability

This study is based on previously published data available in the cited literature. No new datasets were created or analyzed. All data supporting the findings of this review are available within the cited sources.
