# Supplementary material for: A PRISMA-based systematic review on advances in identity recognition and authentication using human biometric signals (2018–2023)
Source: Biomed Eng Online. 2026 Jan 2;25:18. doi: 10.1186/s12938-025-01508-z (PMC12866188; doi:10.1186/s12938-025-01508-z)
Supplement: Supplementary file 1 — Supplementary material 1. [file 12938_2025_1508_MOESM1_ESM.pdf]

Table 14: Complete Search Strategies

Database	Search String	Filters Applied	Date Range	Records Found
PubMed	("biometric authentication" OR "biometric recognition" OR "biometric identification") AND ("ECG" OR "EEG" OR "PPG")	English only	2018–2023	623
Scopus	TITLE-ABS-KEY("biometric authentication" OR "biometric recognition" OR "biometric identification") AND TITLE-ABS-KEY(ECG OR EEG OR PPG)	English; journal articles only	2018–2023	512
IEEE Xplore	("biometric authentication" OR "biometric recognition") AND (ECG OR EEG OR PPG)	Conference papers excluded	2018–2023	412
Web of Science	TS=("biometric authentication" OR "biometric recognition" OR "biometric identification") AND TS=(ECG OR EEG OR PPG)	English; journal type only	2018–2023	331
EBSCO	("biometric authentication" OR "biometric recognition" OR "biometric identification") AND (ECG OR EEG OR PPG)	Peer-reviewed only	2018–2023	186

Note: Search queries were executed in December 2023 across five primary databases. Boolean operators (AND, OR) were used consistently, and search limits were applied to English-language publications from 2018 to 2023.

- The data were obtained from the open-source data sharing platform Mendeley (Relevant articles; [67]).
- The data were obtained from the open-source data sharing platform ieee dataport (Relevant articles; [65]).
- The data were obtained from the open-source data sharing platform UC Irvine (Relevant articles; [86]).
- The data were obtained from the open-source data sharing platform borealisdata (Relevant articles; [74, 10]).
- The data were obtained from the open-source data sharing platform figshare (Relevant articles; [74, 56, 96, 39, 79]).
- The data were obtained from the open-source data sharing platform paperscode (Relevant articles; [65, 88, 82, 80, 36, 57]).
- The data were obtained from the open-source data sharing platform zenodo (Relevant articles; [69, 22, 73, 31, 47, 38, 98, 33, 63, 50, 94, 95, 99]).
- The data were obtained from the open-source data sharing platform physionet (Relevant articles; [67, 65, 88, 82, 80, 36, 57, 74, 45, 37, 48, 37, 37, 74, 55, 41, 91, 74, 41, 91, 44, 74, 56, 44, 78, 77, 44, 67, 15, 74, 74, 90, 56, 74, 71, 87, 59, 41, 91, 56, 15, 57, 36, 65, 30, 28, 43, 74, 56, 114, 86, 83]).
- FVC2004, Link for the dataset, http://bias.csr.unibo.it/fvc2004 (Relevant articles; [99]).
- UBIRIS, Link for the dataset, http://iris.di.ubi.pt/ (Relevant articles; [99]).
- RSVP, Link for the dataset, https://bci.med.tsinghua.edu.cn/download.html (Relevant articles; [99]).

Table 15: JBI Quality Assessment — Ranked Studies (1–40)

No.	Title	References	Subjects	Signals	Accuracy (%)	JBI	Quality
1	A Deep Learning Technique for Biometric Authentication Using ECG Beat Template Matching	[22]	109	EEG	99.98	5	Low
2	A driver authentication system integrated to stress-level determination for driving safety	[27]	18	ECG	93.14	2	Low
3	A Dual Multimodal Biometric Authentication System Based on WOA-ANN and SSA-DBN Techniques	[30]	460	ECG	97.50	2	Low
4	A Low-Complexity Compressed Sensing Reconstruction Method for Heart Signal Biometric Recognition	[29]	55	ECG	99.30	3	Low
5	A Robust Biometric Authentication System ...	[24]	150	ECG	99.80	5	Low
6	A Wearable Wrist Band-Type System ...	[28]	460	ECG	98.40	5	Low
7	An Efficient Optimized Feature Selection ...	[23]	42	EEG	97.60	2	Low
8	Arrangements of Resting State EEG ...	[26]	45	EEG	94.27	3	Low
9	Biometric Authentication from Photic Stimulated EEG Records	[25]	200	PPG	99.00	4	Low
10	Biometric Identification system using EEG signals	[99]	109	EEG	99.00	5	Low
11	Biometric Authentication Using the PPG ...	[39]	47	ECG	95.46	3	Low
12	Biometric person authentication framework ...	[36]	390	ECG	98.10	3	Low
13	Biosignal classification for human identification ...	[37]	183	ECG	98.31	5	Low
14	Cancelable electrocardiogram biometric system ...	[33]	109	EEG	83.21	2	Low
15	Channel Reduction for an EEG-Based Authentication System ...	[38]	109	EEG	98.80	4	Low
16	Classification of ECG signals using multi-cumulants ...	[35]	58	EEG	98.78	4	Low
17	Collaborative-Set Measurement for ECG-Based Human Identification	[34]	23	PPG	95.65	2	Low
18	Combining Cryptography with EEG Biometrics	[42]	22	ECG	94.16	2	Low
19	Compressed-Domain ECG-Based Biometric User Identification ...	[43]	1985	ECG	92.00	4	Low
20	Convolutional Neural Network for Closed-Set Identification ...	[44]	200	ECG	100.00	4	Low
21	Convolutional Neural Network for Individual Identification ...	[48]	20	ECG	99.13	4	Low
22	Data Improvement Model Based on ECG Biometric ...	[45]	50	ECG	99.80	5	Low
23	Driver Identification System Using Normalized ECG ...	[41]	400	ECG	100.00	5	Low
24	ECG Biometrics Using Deep Learning and Relative Score ...	[46]	100	ECG	98.00	6	High
25	ECG Authentication Based on Non-Linear Normalization ...	[40]	127	PPG	99.70	4	Low
26	ECG Classification system based on multi-domain features ...	[49]	20	EEG	88.00	1	Low
27	ECG data optimization for biometric human recognition ...	[50]	109	EEG	99.00	4	Low
28	ECG Identification For Personal Authentication Using LSTM ...	[60]	11	ECG	87.61	2	Low
29	EEG Channel Selection Using Multiobjective Cuckoo Search ...	[55]	23	ECG	91.30	4	Low
30	EEG electrode selection for person identification ...	[59]	90	ECG	99.05	4	Low
31	EEG Authentication System Based on One- and Multi-Class ...	[62]	20	ECG	99.00	4	Low
32	EEG authentication system using fuzzy vault scheme	[67]	290	ECG	99.27	4	Low
33	EEG-Based Biometric Authentication Using Gamma Band ...	[56]	200	ECG	98.00	4	Low
34	EEG-Based Identity Authentication Framework Using Face ...	[57]	290	ECG	99.90	4	Low
35	EEG-Based Person Identification and Authentication ...	[65]	290	ECG	100.00	5	Low
36	Electrocardiogram (ECG)-Based User Authentication Using DL	[54]	20	EEG	98.50	5	Low
37	Efficiently Updating ECG-Based Biometric Authentication ...	[63]	109	EEG	97.00	4	Low
38	Electrocardiogram Biometrics Using Transformer's ...	[52]	35	EEG	99.10	5	Low
39	Electrocardiogram-Based Biometric Identification Using Mixed Feature Extraction	[68]	35	PPG	99.50	2	Low
40	Electrocardiograph Identification Using Hybrid Quantization ...	[64]	35	PPG	97.00	2	Low

Notes: Accuracy reported as percentage; JBI score per itemized checklist (0–8). "Quality" labels follow the thresholding scheme defined in Methods (Sec. 2.7). Abbreviations: ECG, EEG, PPG, etc., are standardized across tables.

Table 16: JBI Quality Assessment — Ranked Studies (41–80)

No.	Title	References	Subjects	Signals	Accuracy (%)	JBI	Quality
41	Electroencephalogram-Based Subject Matching Learning ...	[53]	127	PPG	99.69	5	Low
42	Electroencephalography Feature Enhancement Based on ...	[61]	4	sEMG	93.10	1	Low
43	E-safe: Smart Ecg-based Authentication On-wrist ...	[74]	500	ECG	98.00	2	Low
44	Expression-EEG Bimodal Fusion Emotion Recognition ...	[71]	90	ECG	94.00	2	Low
45	Face Biometric Spoof Detection Method Using a Remote ...	[84]	18	ECG	99.87	3	Low
46	Fusion of Neuro-Signals and Dynamic Signatures ...	[76]	17	ECG	100.00	3	Low
47	Human Identification by Cross-Correlation and Pattern ...	[77]	63	ECG	98.42	3	Low
48	Hybrid Deep Learning and Discrete Wavelet Transform ...	[78]	50	ECG	99.30	3	Low
49	Identity Recognition in Sanitary Facilities ...	[79]	47	ECG	95.17	3	Low
50	Individual Biometric Identification Using Multi-Cycle ...	[82]	290	ECG	95.30	4	Low
51	Initial Study Using Electrocardiogram for Authentication ...	[81]	3133	ECG	99.60	4	Low
52	Intelligent Deep Models Based on Scalograms of ECG ...	[51]	90	ECG	98.60	6	High
53	Investigation of EEG-Based Biometric Identification ...	[85]	21	EEG	86.74	1	Low
54	Leveraging Multiple Distinct EEG Training Sessions ...	[13]	12	EEG	83.15	2	Low
55	Locomotion Mode Recognition for Walking on Three Terrains ...	[69]	109	EEG	93.86	2	Low
56	Multifeature Deep Cascaded Learning for PPG Biometric ...	[83]	9	EEG	96.00	2	Low
57	Multimodal biometric authentication based on deep fusion ...	[73]	109	EEG	98.54	3	Low
58	Novel Biometric Approach Based on Diaphragmatic ...	[98]	109	EEG	99.00	4	Low
59	On the Impact of the Data Acquisition Protocol on ECG ...	[31]	21	EEG	99.00	4	Low
60	Perspectives of human verification via binary QRS ...	[70]	127	PPG	99.75	6	High
61	Phase Portrait of Electrocardiogram as a Means Of Biometry	[72]	127	PPG	99.88	7	High
62	Photoplethysmogram Biometric Authentication Using a 1D ...	[75]	17466	rPPG	99.74	6	High
63	Photoplethysmography Biometric Recognition Model ...	[90]	79	ECG	99.14	2	Low
64	PPG biometric recognition with singular value ...	[87]	90	ECG	99.85	3	Low
65	Recognition System Using Fusion Normalization ...	[88]	115	ECG	99.00	3	Low
66	ResNet-Attention model for human authentication using ECG	[10]	18	ECG	100.00	4	Low
67	SaS-BCI: a new strategy to predict image memorability ...	[96]	47	ECG	99.00	4	Low
68	Selection of the Minimum Number of EEG Sensors ...	[80]	290	ECG	99.69	4	Low
69	Self-Relative Evaluation Framework for EEG-Based ...	[91]	156	ECG	99.62	4	Low
70	Statistical n-Best AFD-Based Sparse Representation ...	[15]	90	ECG	99.89	5	Low
71	Study on PPG Biometric Recognition Based on Multifeature ...	[89]	39	EEG	91.10	2	Low
72	System on Chip (SoC) for Invisible Electrocardiography ...	[86]	139	EEG	99.00	3	Low
73	Task sensitivity in EEG biometric recognition	[92]	29	EEG	96.70	3	Low
74	The Identification of ECG Signals Using WT-UKF and IPSO-SVM	[95]	109	EEG	100.00	5	Low
75	Towards a minimal EEG channel array for a biometric ...	[94]	109	EEG	100.00	5	Low
76	Transcending Conventional Biometry Frontiers: Diffusive ...	[97]	8	EMG	96.00	1	Low
77	Using Convolutional Neural Network and a Single Heartbeat	[93]	35	PPG	99.00	2	Low
78	ECG Signal as Robust and Reliable Biometric Marker ...	[47]	109	EEG	99.30	5	Low
79	ECG-Based Authentication Using Timing-Aware ...	[58]	100	ECG	95.40	3	Low
80	EEG-Based Person Identification during Escalating Cognitive Load	[66]	16	EEG	97.17	2	Low

Notes: Accuracy is reported as percentage. JBI score per itemized checklist (0–8), quality labels per Methods (Sec. 2.7). Abbreviations (ECG, EEG, PPG, rPPG, EMG, sEMG) are standardized across the manuscript.

Table 17: PRISMA 2020 Checklist

Section	Topic	Checklist Item	Location in Manuscript
Title	Title	Identify the report as a systematic review.	Title Page
Abstract	Abstract	Provide a structured summary including objectives, methods, results, and conclusions.	Abstract
Introduction	Introduction	Describe the rationale for the review in the context of existing knowledge.	Introduction (Sec. 1.1)
Methods	Eligibility criteria	Specify the inclusion and exclusion criteria for the review.	Methods (Sec. 2.2)
Methods	Information sources	Specify all databases and information sources searched.	Methods (Sec. 2.1)
Methods	Search strategy	Present the full search strategies for all databases.	Supplementary S1
Methods	Selection process	State the process for selecting studies.	Methods (Sec. 2.2)
Methods	Data collection process	Describe the methods for data extraction from included studies.	Methods (Sec. 2.3)
Methods	Data items	List and define all variables and outcomes sought.	Methods (Sec. 2.4)
Methods	Study risk of bias assessment	Specify methods used to assess risk of bias of included studies.	Methods (Sec. 2.7)
Results	Study selection	Report numbers of studies screened, assessed, and included, with reasons for exclusions.	Results (Sec. 3.1; PRISMA Flow)
Results	Study characteristics	Present characteristics for each included study.	Results (Sec. 3.1; Tables)
Results	Risk of bias in studies	Present assessments of risk of bias for each study.	Results (Sec. 3.8)
Results	Results of individual studies	For all outcomes, present results of each study and summary data.	Results (Sec. 3.9; Tables)
Discussion	Discussion	Summarize key results, limitations, conclusions, and implications.	Discussion (Sec. 4)
Other Information	Registration and protocol	Provide registration information, if registered, or state that the review was not registered.	Not registered
Other Information	Support	Describe sources of support for the review.	Acknowledgments
Other Information	Competing interests	Declare any competing interests of review authors.	Conflict of Interest
Other Information	Availability of data	Report where the review data, materials, and code can be accessed.	Data Availability

Note: Checklist items are mapped to manuscript sections according to PRISMA 2020 guidance; page/line references can be added after final pagination.

- MWM-HIT ECG, Link for the dataset„ https://code.google.com/archive/p/hit-mw-database/ (Relevant articles; [35]).
- MCYT Baseline Corpus, Link for the dataset, https://github.com/luizgh (Relevant articles; [35]).
- MMCBNU-6000, Link for the dataset, https://huggingface.co/luyu0311 (Relevant articles; [125]).
- MAHNOB-HCI, Link for the dataset, https://mahnob-db.eu/ (Relevant articles; [99]).
- Sternberg Task, Link for the dataset, https://openneuro.org/ (Relevant articles; [40], [53], [72], [70]).
- SBVPI dataset, Link for the dataset, https://sclera.fri.uni-lj.si/datasets.html (Relevant articles; [25]).
- VeinPolyU Finger Vein, Link for the dataset, https://web.comp.polyu.edu.hk/csajaykr/ (Relevant articles; [40], [53], [72], [70]).
- Schiller ECG, Link for the dataset, https://www.ncbi.nlm.nih.gov/pmc/articles/PMC5957345/ (Relevant articles; [93], [64], [68]).
- No source could be found for the CuECG and Averio ECG datasets (Relevant articles; [25, 32, 36]).
